# Neonatal Glanzmann's Thrombasthenia Presenting as Refractory Post-circumcision Hemorrhage in a Region of High Consanguinity: A Case Report

**DOI:** 10.7759/cureus.103211

**Published:** 2026-02-08

**Authors:** Nasher H Alyami, Sarah H Musallam, Hasan Al Greshah, Hussain Hajjaf, Salem Alshuqayh, Ali AlAlhareth, Anwar Al Abdali, Jaffar Almakrami, Rashed Almunajem

**Affiliations:** 1 Laboratory Medicine Department, Hematology Unit, Najran General Hospital, Najran, SAU; 2 Internal Medicine Department, King Khalid Hospital, Najran, SAU; 3 Pediatrics Department, Maternity and Children Hospital, Najran, SAU; 4 Laboratory Medicine Department, Maternity and Children Hospital, Najran, SAU; 5 Internal Medicine Department, Maternity and Children Hospital, Najran, SAU; 6 Radiology Department, King Khalid Hospital, Najran, SAU

**Keywords:** case report, circumcision, consanguinity, flow cytometry, glanzmann thrombasthenia, neonatal hemorrhage, platelet glycoprotein iib-iiia complex

## Abstract

Glanzmann's thrombasthenia (GT) is a rare autosomal recessive disorder characterized by deficient or dysfunctional platelet glycoprotein IIb/IIIa (αIIbβ3 integrin), the principal fibrinogen receptor. In neonates, GT is often clinically silent until unmasked by a hemostatic challenge such as surgery or trauma. Standard coagulation screens are normal, making timely diagnosis difficult and potentially leading to life-threatening complications. We report the case of a seven-day-old Saudi boy from Najran who developed severe, refractory hemorrhage following circumcision. Initial surgical hemostasis failed despite normal platelet count and coagulation studies. Flow cytometry revealed a diagnostic immunophenotype for GT with discordant glycoprotein expression: CD41 (GPIIb) at 29% and CD61 (GPIIIa) at 7%. A retrospective review identified significant risk factors: first-degree parental consanguinity and a family history of a bleeding disorder, which had not been assessed pre-procedure. The infant was stabilized with packed red blood cells, fresh frozen plasma, and recombinant activated factor VIIa (rFVIIa). At a two-month follow-up, he remained well with only minor, self-limiting mucocutaneous bleeding. In conclusion, this case demonstrates that refractory post-procedural bleeding in neonates, despite normal routine labs, necessitates prompt investigation for a qualitative platelet disorder such as GT or other inherited thrombocytopathies. In regions with high consanguinity, where local registries report a markedly elevated prevalence, systematic preoperative screening for family bleeding history is a critical preventive measure. Flow cytometry provides a rapid preliminary diagnosis to guide acute management, though definitive GT subtyping requires platelet function studies and genetic confirmation.

## Introduction

Glanzmann's thrombasthenia (GT) is a rare, autosomal recessive inherited platelet function disorder caused by quantitative or qualitative defects in the glycoprotein IIb/IIIa complex (integrin αIIbβ3) [1]. This receptor, which serves as the principal fibrinogen receptor on platelets, is essential for platelet aggregation and primary hemostasis, mediating fibrinogen binding and clot formation. The global prevalence of GT is estimated at approximately one in 1,000,000, but this figure is substantially elevated in populations with high rates of consanguineous marriage, such as in the Middle East, South Asia, and among specific ethnic groups [2-6]. In regions such as Saudi Arabia, local registries report a prevalence as high as approximately one in 10,000, highlighting the significant impact of consanguinity [3].

Clinical manifestations typically involve mucocutaneous and post-surgical bleeding, including purpura, epistaxis, gingival bleeding, and menorrhagia. The neonatal presentation is uncommon and can be diagnostically challenging. Signs may include unexplained bruising, cephalohematoma, umbilical stump oozing, or, as highlighted in this report, severe hemorrhage following minor surgical procedures such as circumcision [7]. Diagnosis is frequently delayed because the first-line laboratory investigations, platelet count, prothrombin time (PT), and activated partial thromboplastin time (aPTT) are invariably normal [7,8].

Confirmatory diagnosis relies on specialized testing. Light transmission aggregometry, demonstrating absent aggregation in response to agonists such as adenosine diphosphate (ADP) and collagen but normal response to ristocetin, remains the diagnostic gold standard. However, flow cytometry has emerged as a rapid and highly useful alternative, particularly in urgent clinical scenarios or resource-limited settings where aggregometry is unavailable [9]. Quantifying the surface expression of CD41 (GPIIb) and CD61 (GPIIIa) on platelets can confirm the diagnosis even with the small blood volumes obtainable from neonates [1,9]. In neonates, common hemostatic challenges, such as minor surgical procedures (e.g., circumcision), may unmask underlying platelet function defects, leading to persistent or excessive bleeding [2,10].

In regions with a high prevalence of consanguinity, a proactive approach involving a detailed family assessment and genetic counseling is crucial for risk stratification and perioperative planning. This case report details the presentation, diagnostic journey, and management of early-onset GT in a Saudi neonate, underscoring the pivotal role of this family assessment and the utility of flow cytometry in enabling a timely, life-saving diagnosis.

## Case presentation

A seven-day-old full-term male neonate (gestational age: 39 weeks; birth weight: 3.0 kg; Apgar scores: 9 and 10 at one and five minutes, respectively) was urgently referred to the Emergency Department of the Maternity and Children Hospital (MCH) in Najran, Saudi Arabia, for persistent, active bleeding from a circumcision site. The procedure had been performed approximately four hours prior at a local private clinic. No analgesics or anesthetics were administered during the procedure, as per the parents' report. Initial attempts at hemostasis with direct pressure and gauze dressings at the clinic were unsuccessful. The infant was born via an uncomplicated vaginal delivery to first-degree consanguineous parents. A maternal family history of a female cousin with factor VII deficiency was noted, but no prior comprehensive genetic screening had been performed. The patient's three elder siblings (from the same parents) were healthy, with no history of bleeding disorders. Vitamin K prophylaxis had been administered at birth according to standard hospital protocol.

On admission, the infant was jaundiced with clinical scleral icterus but was otherwise alert, active, and afebrile. Physical examination revealed a well-nourished neonate with continuous oozing from both the ventral and dorsal aspects of the penile surgical wound, leading to a significant drop in hemoglobin (8.4 g/dL), indicating acute blood loss. No petechiae, ecchymoses, hepatosplenomegaly, or other systemic abnormalities were detected.

Immediate local hemostatic measures were undertaken, including bipolar electrocautery and compressive “blanket” suturing, which achieved temporary hemostasis. However, significant bleeding recurred on postoperative day two following dressing removal. Initial laboratory evaluation (Table 1) revealed a normocytic, normochromic anemia (hemoglobin: 8.4 g/dL) secondary to acute blood loss, with a normal platelet count (323 × 109/L) and white blood cell count (12.1 × 109/L), and standard coagulation parameters (PT: 12.8 s, aPTT: 31.0 s). Review of the peripheral blood smear revealed normal platelet morphology. The template bleeding time was markedly prolonged at 7.4 minutes.

**Table 1:** Initial laboratory findings on admission *Note: Elevated total bilirubin was consistent with physiological jaundice of the newborn; no laboratory evidence of hemolysis was present.

Investigation	Result	Reference Range
Hemoglobin (Hb)	8.4 g/dL	13.5-18.5 g/dL
White Blood Cell Count	12.1 × 10⁹/L	5.0-21.0 × 10⁹/L
Platelet Count	323 × 10⁹/L	150-450 × 10⁹/L
Platelet Morphology (Peripheral Smear)	Normal	Normal
Prothrombin Time (PT)	12.8 s	11.0-13.5 s
Activated Partial Thromboplastin Time (aPTT)	31.0 s	25.0-35.0 s
Bleeding Time (Template)	7.4 min	2.0-5.0 min
Total Bilirubin*	137 μmol/L	< 85 μmol/L (day 7)

The anemia was corrected with a transfusion of 15 mL/kg of packed red blood cells. Given the clinical scenario of refractory mucocutaneous bleeding with normal platelet counts and coagulation times, a qualitative platelet function disorder was strongly suspected. Other potential causes, such as von Willebrand disease (where PT/aPTT are also often normal) or factor XIII deficiency, were considered. However, the markedly prolonged bleeding time and the immediate context of a surgical challenge pointed more strongly toward a primary platelet disorder.

Platelet light transmission aggregometry was not available locally. Therefore, diagnostic flow cytometry for platelet surface glycoproteins was performed on a peripheral blood sample. The results demonstrated a normal expression of CD42b (GPIb) at 96% (reference: >70%), but significantly reduced expression of the αIIbβ3 integrin components: CD41 (GPIIb) at 29% and CD61 (GPIIIa) at 7% (reference for both >70%). The preserved CD42b effectively ruled out Bernard-Soulier syndrome.

This immunophenotypic pattern is diagnostic for GT. However, the expression levels were discordant: CD61 (7%) falls within the classic range for type II GT (5-20% of normal), while CD41 (29%) is above this range, more consistent with a variant or partial expression phenotype. This discordance suggests a potential mutation differentially affecting the stability, assembly, or surface trafficking of the αIIb (GPIIb) versus β3 (GPIIIa) subunits. Therefore, while the diagnosis of GT is confirmed, definitive subclassification into type I, type II, or variant GT cannot be made based on flow cytometry alone and requires correlative functional studies (platelet aggregometry, clot retraction) and molecular genetic sequencing of the ITGA2B and ITGB3 genes.

Definitive control of bleeding was achieved using a combination of fresh frozen plasma (FFP), administered at 10 mL/kg, and recombinant activated factor VII (rFVIIa) at a dose of 90 µg/kg. The infant was closely monitored for potential thrombotic complications of rFVIIa, and none were observed during the hospital stay. Platelet transfusion was not employed as first-line therapy in this diagnosed case due to the rapid and effective hemostasis achieved with rFVIIa and to mitigate the risk of early alloimmunization against platelet glycoproteins, a common complication in GT.

**Figure 1:**
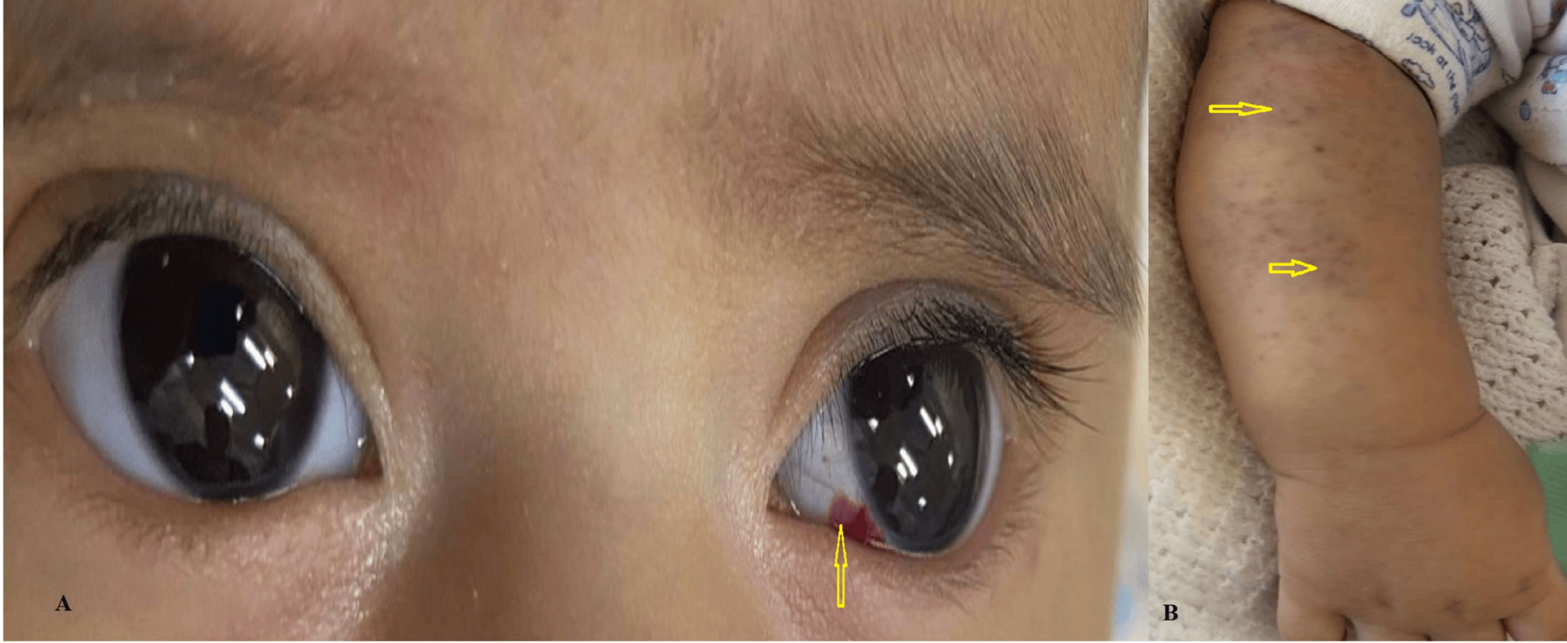
Mucocutaneous bleeding manifestations at two-month follow-up (A) Close-up photograph of the patient's right eye showing a localized subconjunctival hemorrhage, a hallmark of qualitative platelet dysfunction. (B) Clinical photograph of the patient's right forearm demonstrating numerous petechiae and minor purpura. These recurring, self-limiting mucocutaneous bleeds are characteristic of the clinical course following the stabilization of the initial severe post-circumcision hemorrhage.

The infant's condition stabilized, and he was discharged on hospital day 12. The parents received extensive counseling on bleeding precautions, including the avoidance of intramuscular injections, non-steroidal anti-inflammatory drugs (NSAIDs), aspirin, and contact sports. An emergency alert card was provided. At a two-month follow-up, the infant was thriving with only minor, self-limiting mucocutaneous bleeding (mild purpura, a small subconjunctival hemorrhage) (Figure 1). 

The family has been referred for formal genetic counseling, and plans are in place for platelet function testing and ITGA2B/ITGB3 gene sequencing at a tertiary hematology center.

## Discussion

This case highlights the significant diagnostic challenges inherent in identifying congenital platelet disorders in the neonatal period. A routine elective procedure, circumcision, acted as a hemostatic challenge, unmasking a severe bleeding diathesis. Retrospectively, the presence of first-degree parental consanguinity and a family history of a coagulation disorder (factor VII deficiency in a cousin) were substantial risk indicators. While the factor VII deficiency in a maternal cousin may be a coincidental finding, it nonetheless signals a family pedigree with a potential burden of bleeding disorders, reinforcing the importance of a detailed family history. Integrating these details into the preoperative assessment could have prompted a hematology consultation and prevented the surgical complication altogether.

While light transmission aggregometry remains the diagnostic gold standard for platelet function disorders, its limited availability in non-specialized centers is a practical reality [9]. In this context, flow cytometry served as a critical diagnostic tool, particularly helpful as aggregometry was unavailable. It rapidly identified the characteristic immunophenotype of GT: preserved CD42b (GPIb) expression (96%) with significantly reduced expression of the αIIbβ3 integrin - the principal fibrinogen receptor on platelets. However, the pattern was discordant, with CD61 (GPIIIa) at 7% and CD41 (GPIIb) at 29%. The discordant CD41 (29%) and CD61 (7%) expression observed in our patient is noteworthy. Classic type II GT typically demonstrates a concordant reduction of both CD41 and CD61 to within 5-20% of normal, whereas type I shows near-absent expression (<5%), and variant forms exhibit near-normal levels with qualitative dysfunction [9,11]. The observed pattern, with CD61 in the type II range but CD41 elevated above it, suggests an underlying genetic defect that differentially affects β3 subunit expression or the assembly and trafficking of the αIIbβ3 complex. Such discordant phenotypes have been documented in the literature and underscore the necessity of molecular genetic analysis of ITGA2B and ITGB3 for definitive subclassification and to understand the specific pathophysiologic mechanism [1,9,11]. Flow cytometry thus provides crucial diagnostic and preliminary subtyping information, though it cannot replace definitive functional and genetic studies.

To control the acute hemorrhage, hemostasis was secured using rFVIIa and FFP. Although platelet transfusion is considered first-line therapy for major bleeding in GT [12], it was withheld initially in this case. This decision was based on the rapid achievement of hemostasis with rFVIIa/FFP and the desire to mitigate the risk of early alloimmunization against platelet glycoproteins, a common and management-complicating consequence in GT patients who receive repeated transfusions [12,13]. rFVIIa promotes hemostasis via a tissue factor-dependent, platelet-independent pathway, making it a valuable agent in GT, particularly in settings of refractoriness or to avoid sensitization [13,14]. Its successful use in neonatal GT, as in our case, is supported by several case reports and reviews [7,15]. The infant was closely monitored for potential thrombotic complications of rFVIIa, and none were observed during the hospital stay.

The clinical context of this case is inextricably linked to regional demographics. Consanguineous marriage rates in Saudi Arabia are among the highest globally, reported to range from 40% to 60% in various studies [4-6]. This practice significantly elevates the carrier frequency and birth prevalence of autosomal recessive disorders such as GT compared to outbred populations. While precise, population-based prevalence data for GT in Saudi Arabia are lacking, it is well-established that the disorder is markedly more common in regions with high consanguinity than the estimated global prevalence of approximately one in 1,000,000, with local registries reporting a prevalence as high as approximately one in 10,000 [2,3]. Standard preoperative coagulation screens (PT/aPTT) are entirely normal in qualitative platelet disorders, creating a dangerous diagnostic blind spot. This reality underscores the critical need to enhance neonatal care protocols in high-risk regions. Implementing a simple, mandatory pre-procedural checklist to document consanguinity and a detailed family assessment would efficiently identify neonates warranting further specialized hematological evaluation before elective surgery, thereby preventing hemorrhagic complications.

While initial light transmission aggregometry and definitive genetic sequencing were not available at the time of presentation, the immunophenotypic profile and clinical course were highly suggestive of GT. The absence of platelet aggregation studies and genetic testing prevents definitive GT subtype classification, and the discordant CD41/CD61 pattern observed in our patient requires molecular confirmation to identify the specific underlying mutation. These studies are planned for this patient and will provide important information for genetic counseling and family screening. The next step for regional maternal and child health centers should be the implementation and validation of family assessment tools to identify at-risk neonates before they reach the operating table. Mandatory screening for consanguinity and a detailed bleeding history, potentially operationalized through a simple checklist embedded in pre-procedural consent forms, should be integrated. Future research should focus on establishing standardized preoperative screening protocols for high-risk populations and evaluating their cost-effectiveness in preventing hemorrhagic complications.

## Conclusions

In summary, this case underscores that refractory post-procedural bleeding in neonates with normal routine coagulation studies necessitates prompt evaluation for qualitative platelet disorders. Flow cytometry provides a rapid and reliable preliminary diagnosis in such emergencies and assists in preliminary subtype assignment, though it cannot replace definitive functional and genetic studies. A thorough, systematic family assessment remains the most cost-effective preventive tool. Therefore, mandatory preoperative screening for consanguinity and bleeding history, potentially operationalized through a checklist embedded in consent forms, should be integrated into standard protocols in high-prevalence regions. A proactive, preventative model is superior to reactive crisis management. Ultimately, while flow cytometry is diagnostically powerful, its findings should be confirmed with advanced studies to guide definitive diagnosis and long-term care for conditions such as GT.
